# Supplementary material for: Ethical inclusion: Risks and benefits of research from the perspective of perinatal people with opioid use disorders who have experienced incarceration
Source: PLoS One. 2023 Nov 22;18(11):e0294604. doi: 10.1371/journal.pone.0294604 (PMC10664874; doi:10.1371/journal.pone.0294604)
Supplement: S1 File — (PDF) [file pone.0294604.s001.pdf]

University of North Carolina at Chapel Hill
Consent to Participate in a Research Study
Pregnant and Postpartum Participants

Consent Form Version Date: Oct. 13, 2021
IRB Study # 20-3559
Title of Study: MATernity Cohort Study: Interviews to inform recruitment and retention protocols
Principal Investigator: Andrea Knittel
Principal Investigator Department: Obstetrics and Gynecology-General Obstetrics and Gynecology
Principal Investigator Phone number: (919) 843-7851
Principal Investigator Email Address: andrea_knittel@med.unc.edu
Funding Source and/or Sponsor: NIH National Institute of Child Health and Human Development (NICHD)

Study Contact Telephone Number: (919) 429-9173
Study Contact Email: maternity@unc.edu

CONCISE SUMMARY

The goal of this study is to learn about the challenges facing women who use drugs and are pregnant or just had a baby while they are in prison. We want to understand what makes women decide to be in a research study or not to be in a research study, and what might make it easier for them to stay in a study when they go home. We are interviewing 15 women who are either currently in prison or were recently in jail or prison and had the experience of being pregnant, giving birth, or were postpartum while there. If you decide to participate, your interview will take 60-90 minutes. You will be given codes to give to family members or the person who will take care of your baby so they can decide if they want to be interviewed. The only benefit of participating is the chance to talk to someone about your experiences. The risks of participating include feeling embarrassed while discussing drug use or being in prison, and a very small risk that someone could learn that you participated in the study.

What are some general things you should know about research studies?

You are being asked to take part in a research study. To join the study is voluntary. Participating will not affect your medical care or your prison time.

You may choose not to participate, or you may withdraw your consent to be in the study, for any reason, without penalty. Deciding not to participate or ending your participation early will not affect your medical care or your prison time.

Research studies are designed to obtain new knowledge. This new information may help people in the future. You may not receive any direct benefit from being in the research study. There also may be risks to being in research studies.

Details about this study are discussed below. It is important that you understand this information so that you can make an informed choice about being in this research study.

You will be given a copy of this consent form. You should ask the researchers named above, or staff members who may assist them, any questions you have about this study at any time.

What is the purpose of this study?
The goal of this study is to learn about the challenges facing women who use drugs and are pregnant or just had a baby while they are in prison. We want to understand what makes women decide to be in a research study or not to be in a research study, and what might make it easier for them to stay in a study when they go home.

You are being asked to be in the study because you are pregnant or just had a baby while you are in prison and because you have used drugs in the past.

Are there any reasons you should not be in this study?
You should not be in this study if it would make you too uncomfortable to talk about pregnancy, drug use, and being in prison.

How many people will take part in this study?
There will be approximately 30 total participants. Fifteen (15) people at NCCIW (North Carolina Correctional Institution for Women) will take part in this study. Another 15 family members or infant caregivers will participate outside the prison.

How long will your part in this study last?
The interview will last 60-90 minutes once the consent process is finished. You will be provided with codes to let your family members or individuals who will care for your baby contact the researchers if they want to participate. We expect that it will take 10-15 minutes to share the study information with them.

What will happen if you take part in the study?

- ***Research Interview***
  - Answer questions about pregnancy and the time after birth in prison, drug use, the transition to the community, and research studies.
  - The observer who witnesses the consent (nurse, clinical or non-clinical staff) will stay in the room for the entire interview.
  - You may choose not to answer any question for any reason.
  - You may choose to stop taking part in the study at any time.
  - The prenatal doctors will not know whether you chose to participate in the research or not.

    - Department of Public Safety staff are not conducting this research project. They will not get a copy of your name or of your answers. The Department may receive a copy of the overall results at the end of the study but will not be able to identify you personally from the copy they receive.
    - Your release date, terms of supervision, medical care, or your general living conditions will not be affected by whether you chose to be in the study or if you chose to stop participating at any point.
    - You should know that if you indicate plans to harm yourself, to harm someone else, or to escape or abscond supervision that information is not confidential and will immediately be reported to DPS staff.

- ***Codes for family members or infant caregivers:*** You will receive five unique codes to give to your family members or to the people who will help care for your baby while you are in prison or jail. No one will know whether you chose to share the study information or not with your family members or infant caregivers.

What are the possible benefits from being in this study?
Research is designed to benefit society by gaining new knowledge. You may not benefit personally from taking part in this research.

What are the possible risks or discomforts involved from being in this study?

- Emotional distress during the interview (small risk): You may skip any question or stop the interview at any time. If needed, you can be referred to mental health resources in the prison.
- Embarrassment during the interview (small risk): You may skip any question or stop the interview at any time.
- The observer in the room or others learning about illegal behavior (small risk) or others discovering that you participated in the study (very small risk): We will make every effort to keep research records private. You may skip any question or stop the interview at any time.

There may be uncommon or previously unknown risks. You should report any problems to the researcher.

What if we learn about new findings or information during the study?
You will be given any new information gained during the course of the study that might affect your willingness to continue your participation.

How will information about you be protected?
We will make every effort to keep research records private, including:

- Your name will only be connected to your interview data with a research code. The file with the research codes will be secured with a password on a secure computer server maintained by UNC;

- None of the notes taken during the interview will include your name or anyone else's identifying information;
- Only the PI and immediate research team will have access to the file with the research codes.

Participants will not be identified in any report or publication about this study. We may use de-identified data (for example, quotes without your name attached to them) from this study in future research without additional consent.

Although every effort will be made to keep research records private, there may be times when federal or state law requires the disclosure of such records, including personal information. This is very unlikely, but if disclosure is ever required, UNC-Chapel Hill will take steps allowable by law to protect the privacy of personal information. In some cases, your information in this research study could be reviewed by representatives of the University, research sponsors, or government agencies (for example, the FDA) for purposes such as quality control or safety.

- We will not be recording this interview. Only written and typed notes without your name or identifying information will be collected.
- The file with your name and the research codes will be kept for two years after the study is over.

<u>What is a Certificate of Confidentiality?</u>

This research is covered by a Certificate of Confidentiality. With this Certificate, the researchers may not disclose or use information, documents or biospecimens that may identify you in any federal, state, or local civil, criminal, administrative, legislative, or other proceedings in the United States, for example, if there is a court subpoena, unless you have consented for this use.

The Certificate cannot be used to refuse a request for information from personnel of a federal or state agency that is sponsoring the study for auditing or evaluation purposes or for information that must be disclosed in order to meet the requirements of the federal Food and Drug Administration (FDA).

The Certificate of Confidentiality will not be used to prevent disclosure as required by federal, state, or local law, such as mandatory reporting requirements for child abuse or neglect, disabled adult abuse or neglect, communicable diseases, injuries caused by suspected criminal violence, cancer diagnosis or benign brain or central nervous system tumors or other mandatory reporting requirement under applicable law. The Certificate of Confidentiality will not be used if disclosure is for other scientific research, as allowed by federal regulations protecting research subjects or for any purpose you have consented to in this informed consent document.

You should understand that a Certificate of Confidentiality does not prevent you from voluntarily releasing information about yourself or your involvement in this research. If an insurer, employer, or other person obtains your written consent to receive research information, then the researchers may not use the Certificate to withhold that information.

What if you want to stop before your part in the study is complete?

You can withdraw from this study at any time, without penalty. The investigators also have the right to stop your participation at any time. This could be because you have had an unexpected reaction, or have failed to follow instructions, or because the entire study has been stopped.

If you withdraw or are withdrawn from this study all data collected up until the point of withdrawal will be retained, however no additional information will be collected unless you provide additional written permission for further data collection at the time of your withdrawal.

Will you receive anything for being in this study?
You will not receive anything for being in this study.

What if you have questions about this study?
You have the right to ask, and have answered, any questions you may have about this research. If you have questions about the study, complaints, concerns, or if a research-related injury occurs, you should contact the researchers listed on the first page of this form.

What if you have questions about your rights as a research participant?
All research on human volunteers is reviewed by a committee that works to protect your rights and welfare. If you have questions or concerns about your rights as a research subject, or if you would like to obtain information or offer input, you may contact the Institutional Review Board at 919-966-3113 or by email to IRB_subjects@unc.edu.

Participant's Agreement:

I have read the information provided above. I have asked all the questions I have at this time. I voluntarily agree to participate in this research study.

____ _______________
Signature of Research Participant Date

____
Printed Name of Research Participant

Signature of Research Team Member Obtaining Consent | Date

Printed Name of Research Team Member Obtaining Consent

Signature of Witness if applicable; e.g. literacy issues, visually impaired, physically unable to sign, witness/interpreter for non-English speaking participants using the short form) | Date

Printed Name of Witness
