# Supplementary material for: Ethical inclusion: Risks and benefits of research from the perspective of perinatal people with opioid use disorders who have experienced incarceration
Source: PLoS One. 2023 Nov 22;18(11):e0294604. doi: 10.1371/journal.pone.0294604 (PMC10664874; doi:10.1371/journal.pone.0294604)
Supplement: S2 File — (PDF) [file pone.0294604.s002.pdf]

MATernity Recruitment and Retention Interview Guide

Pregnant and Postpartum Participants

Consent

Demographic questions: age, race, ethnicity, number of children, weeks or months of pregnancy, safekeeper or not, previous incarcerations, duration of remaining incarceration

I want to remind you that this is not recorded, but I will be taking notes throughout the interview. I will not record your name, or anyone else's, to help keep everything private. Even if you slip up and use someone's name, I will not write it down. We will not be able to connect the things you tell us with your identity or anyone that you talk about during the interview. You can skip any questions that you don't want to answer. You can also stop early for any reason.

There are no right answers to any of these questions – we are asking you these questions because you are the expert here, and we want to learn from you.

Are you ready to begin the interview?

Section 1: Pregnancy

We're going to start with some questions about your pregnancy. Remember that we can skip any questions that you don't want to answer.

Tell me about your pregnancy so far. What has made it a hard pregnancy? An easy pregnancy?

How has this pregnancy been different from other pregnancies?

Would you give me a few examples of how your pregnancy experiences are different because you are in the prison?

Have you been on community supervision, or parole, or probation in your pregnancy? What was that like?

Tell me about what kind of treatment for using drugs you received before you were in the prison, if any. Did you want a treatment that you couldn't get? What kept you from getting that treatment?

Tell me about what kind of treatment for using drugs you are getting in the prison, if any. Do you want a treatment that you can't get?

Are there other things about your pregnancy that are important that I haven't talked mentioned?

Section 2. Recruitment

Now I'd like to discuss what made you decide to be a part of this research study. Is it okay to keep going?

What made you decide to be a part of this research study?

Could you talk me through the process of considering whether you wanted to do it or not?

Is there anything that would have changed your decision? Or made you want to do it more?

Section 3: Transition to the community and postpartum

My next questions are about what will happen when you go home from the prison. Is it still alright to keep talking?

Will you have your baby before you go home, or will you go home while you are still pregnant?

If still pregnant:

> What are you most excited about with going home?
>
> Are you going home to the same place you have lived before? Or somewhere else? Why?
>
> What challenges do you think you might have at home? (Probe: Work? Housing? Community supervision?)
>
> Who are the most important support people for you at home? Could you give me an example for each of those people about how they support you?
>
> Do you think people treat women who have been incarcerated differently where you live? Could you give me an example?
>
> What kinds of drug treatment are available for pregnant people where you live? What are your plans for when you go home?
>
> What else is important for me to know about leaving the prison while you are pregnant?

If postpartum:

> What are you most excited about with going home?
>
> Are you going home to the same place you have lived before? Or somewhere else? Why?
>
> What challenges do you think you might have at home?
>
> Who are the most important support people for you at home? Could you give me an example for each of those people about how they support you?
>
> I'm going to list some examples of support some people want or need after they have a baby. You can tell me whether that is something you would want, if someone will support you that way when you go home, and how.
>
> > Help with taking care of the baby
> >
> > Breastfeeding
> >
> > Depression or mood changes after having a baby
> >
> > Learning about parenting with a new baby
> >
> > Recovery from addiction with a new baby
>
> Do you think people treat women who have been incarcerated differently where you live? Could you give me an example?

What kinds of drug treatment are available for people with small babies where you live? What are your plans for when you go home?

What else is important for me to know about leaving the prison after you have a baby?

Section 4: Retention

The last few questions are about participating in research studies after you go home. We are nearly finished!

If we asked you to do another interview at home after you had your baby, would you want to do it?

Could you talk me through the process of considering whether you would want to do it or not?

How would we reach you if you wanted to keep participating after you went home?

What would make it easier to stay in a research study when you go home?

Is there anything else you would like to share about your experiences?

Thank you so much for your time and for sharing your experiences with us.
