# Supplementary material for: Ethical inclusion: Risks and benefits of research from the perspective of perinatal people with opioid use disorders who have experienced incarceration
Source: PLoS One. 2023 Nov 22;18(11):e0294604. doi: 10.1371/journal.pone.0294604 (PMC10664874; doi:10.1371/journal.pone.0294604)
Supplement: Attachment — Submitted filename: Response to Reviewers.docx [file pone.0294604.s004.docx]

**Response to Reviewers**

**October 20, 2023**

**Comment	Response	Edited Selection**

| Reviewer #1:  The first paragraph of the manuscript reads as overly broad and I'm not sure that it is necessary to start the paper. If the authors choose to retain this paragraph, then there should probably be citations included for the first and second sentences in this paragraph -- if this is "common knowledge" then I'm not quite sure it's necessary to include any of this text to start the paper. | Ah, you are correct. The information in this paragraph is stated, more specifically and with citations, later in the Introduction. The intention was to create a summary “here’s what this paper is”, but you are correct that it is overly broad and redundant with the abstract. We have removed. | Lines 65-71 removed. |
| --- | --- | --- |
| Please spell out United States and give the abbreviation and then use the abbreviation from that point forward consistently. | Thank you! Yes, we have made these corrections throughout. | Fixed on Line 44; 96; 105; 126. We also corrected our abbreviation of North Carolina on lines 44 and 142. |
| Check grammar/sentence structure of the first sentence under eligibility and consent (p. 5, line 131) | Thank you; yes, this was oddly phrased. We have removed ‘currently’ as ‘past year’ encapsulates that timeframe. Hopefully this helps. | “We recruited people who were at least 18 years old, met the clinical criteria for opioid use disorder (OUD), and had experienced perinatal incarceration in the past year.” |
| Two subheadings now refer to consent (pp. 5 & 6). I would remove 'consent' from p. 5 (line 130). | Good catch! We have removed. Thank you | “Consent” removed as section heading (page 5; line 130) |
| The sentence re: payment feels out of place (p. 6, line 158-160) because participants would be compensated after they consented. I would move this sentence to the last sentence before analysis (p. 8). | Great suggestion! We have moved that sentence to the end of the “Interviews and Safety Monitoring” section | Sentence moved to line 199-201. |
| On Page 8, lines 190-191 -- I think you need to make it clear that the main objective of the interview questions that *were used in this study* were to inform robust recruitment and community retention processes. It's clear from the Appendix B that there were other questions included that were there for other purposes (and not presented in this paper). I would just try to make that clear that for *this* paper/study, you pulled out specific questions (Sections 2 & 4, specifically) from the broader interview protocol. | This is a good distinction. Our Primary Study Aim was recruitment and retention, however, we did have subaims, which are reflected in the other research questions. Your suggestion is the most integrous to the interview protocol, therefore we have added the language you recommend. | Line 192-194: “The main objective of the interview questions that inform this analysis was to inform robust recruitment and community retention processes for people with OUD from a prison prenatal clinic.” |
| There are weird lines that appear throughout the paper and I'm not sure if they're intended to be there (e.g., p. 9, 14, 15). | Oh, those lines! We tried everything to remove them – stubborn page breaks. Anyway, in the Manuscript attachment, I have copied our text over to a clean document and they are (finally) gone. Thank you for noticing that! | They still appear in the Track Changes document, but have been removed from the Manuscript attachment. |
| Reviewer #2: Just to say I have nothing else to add to a review. I really like this paper. I am glad to have been able to support it, and look forward to seeing it in print (and being able to cite it).  I would also like to thank the authors for the positive responses to the review and engagement with it. The changes have made it better.  Do you realise you have written an empirical ethics paper? That may be worth you exploring ... | Thank you so much. Your careful read and thoughtful feedback improved our work; we greatly appreciate that. We do hope it will be a useful resource for those doing this important work. Thank you. |  |
